# Supplementary material for: Machine learning for early prediction of the infection in patients with urinary stone after treatment of holmium laser lithotripsy
Source: PLoS One. 2025 May 16;20(5):e0317584. doi: 10.1371/journal.pone.0317584 (PMC12084040; doi:10.1371/journal.pone.0317584)
Supplement: S1 File — (DOCX) [file pone.0317584.s001.docx]

Data preprocessing:

In this study, age >=60 is considered as 1, otherwise 0. Gender is 1 for male and 0 for female. Preoperative albumin＜40g/L is considered as 1, otherwise 0. If hydronephrosis is present, it is assigned a value of 1, otherwise 0. If patients with hypertension or diabetes or kidney disease related diseases, the corresponding characteristic is considered to be 1, otherwise 0. If there is an asymptomatic bacteriuria before surgery or abnormal situation during the operation, it is 1, otherwise, it is 0. Preoperative infection or asymptomatic bacteriuria was controlled before surgery, the corresponding characteristic is considered to be 1, otherwise 0. If the operation time is >=60min, it is 1, otherwise 0. If the stone location is in the kidney, the value is 1, otherwise, the value is 0. If there is abnormal intraoperative condition, the value is 1, otherwise, the value is 0. If the preoperative urine leukocyte count >=25, the value is 1, otherwise 0. If urine occult blood, the value is 1, otherwise 0.

Technical Details for the Statistical Analysis:

This study used Python 3.7 to accomplish the statistical analysis. miceforest (version 1.3.2), pandas (version 1.19.2) and numpy (version 1.3.2) were used to process raw data and complete factor analysis. We used the package scikit-learn (version 1.3.2) which could be used to bulid SVM, Logistic regression, Random Forest model and so on, XGBoost (version 1.1.0), LightGBM (version 2.2.2) to implement various machine learning algorithms. SHAP (version 0.42.0) and matplotlib (version 3.4.0) were used to draw the figure and discuss the clinical variables.
