# Supplementary material for: Machine learning for early prediction of the infection in patients with urinary stone after treatment of holmium laser lithotripsy
Source: PLoS One. 2025 May 16;20(5):e0317584. doi: 10.1371/journal.pone.0317584 (PMC12084040; doi:10.1371/journal.pone.0317584)
Supplement: Attachment — Submitted filename: Response to Reviewers.docx [file pone.0317584.s003.docx]

Dear Editor and Reviewers,

Thank you so much for your comments concerning our manuscript entitled “Machine learning for early prediction of the infection in patients with urinary stone after treatment of holmium laser lithotripsy” (ID: PONE-D-24-39210). Those comments are all valuable and very helpful for revising and improving our paper, as well as the important guiding significance to our researches. We have studied comments carefully and have made correction which we hope meet with approval.

Response to editor:

Thanks for your suggestions! Data was uploaded as supplementary files.

Reviewer #1:

1. In the first paragraph of Introduction, the authors should not only introduce the prevalence of urinary tract stone, but also the epidemiology of the infection after holmium laser lithotripsy, such as its rate and risk factors.

**Authors' Response**: Thanks for the kind and insightful comments and suggestions! We have re-written this part according to your suggestion. Changes to our manuscript were all highlighted within the document by using red-colored text.

2. In Data preprocessing section of Materials and methods, the variable coding methods can be moved into supplementary file, and it’s better to include only feature engineering methods in this part like data transformation and data extraction, if any.

**Authors' Response**: Thank you very much for your advice. We have removed the variable coding methods into supplementary file.

3. Please give the names and versions of all the packages used in each step of the analyses.

**Authors' Response**: Thank you very much for your advice. We have added the details of packages into supplementary file.

4. Do you have both a validating set and a test set? If not, how to identify the best hyperparameters and also evaluate the model performance using the only one testing set under a 5-CV strategy?

**Authors' Response**: Thank you very much for your advice. We have divided the datasets into 2 parts, one was used for 5-cv training process for the best hyperparameters, the other one was regarded as independent test dataset. Figure 1 was 5-cross validation results. We saved the best model to predict the independent dataset as showed in Figure 2.

5. Usually, in clinical predictive models, PPV is very low even if other performance indexes are relatively high, so do you consider additionally calculate PPV to evaluate your model’s performance?

**Authors' Response:** Thank you very much for your advice. Positive Predictive Value (PPV) is equivalent to precision. We had included the F1 score, which simultaneously evaluated both precision and recall. A high F1 score indicates that the model has both good precision and good recall.

6. In the Discussion, lacking external validation may be one of the limitations.

**Authors' Response:** Thank you very much for your advice. In fact, we have recently collected more data and are also attempting to use our developed model to predict whether patients will get infections after holmium laser lithotripsy surgery in order to give antibiotic intervention. Our model should be further optimized according to more datasets.

Reviewer #2:

1. Content and Structure: The manuscript is well-structured, providing a clear explanation of the problem, methodology, and results. However, the introduction would benefit from further elaboration on the clinical challenges of postoperative infection prediction and how machine learning models can provide a unique solution compared to traditional methods. Additionally, the conclusion should expand on the broader implications of the findings and how they can be applied to real-world clinical settings.
**Authors' Response:** We feel great thanks for your professional review work on our article. We have refined our introduction and the conclusion according to your suggestions. Changes to our manuscript were all highlighted within the document by using red-colored text.

2. Literature Review and Citations: The literature review is comprehensive, but incorporating more recent studies on machine learning applications in medical prediction models would strengthen the manuscript. I recommend adding the following papers:
**Authors' Response:** Thank you very much for your advice. We have added the papers in our study.
3. Technical Clarifications and Suggestions: While the technical aspects of the model are well-described, further clarification on the hyperparameter tuning process for the machine learning models would provide valuable insights. Specifically, more details on how the models were optimized (e.g., grid search, cross-validation) would enhance the reproducibility of the study. Additionally, a more detailed comparison of the different models (e.g., Random Forest, Logistic Regression, XGBoost) in terms of computational efficiency and predictive power could provide a deeper understanding of their strengths and weaknesses in this context.
**Authors' Response:** Thank you very much for your advice. We have added the details of training process into supplementary file. Due to the nature of algorithm, tree algorithms like XGBoost and LightGBM take longer time and consume more power, and Logistic Regression takes the minimum time.
4. Lastly, it would be helpful to discuss potential limitations of the study, such as the single-center nature of the dataset, and how these limitations might affect the generalizability of the model.
**Authors' Response:** Thank you very much for your advice. We have added the discussion in our paper in the Discussion part.

Yours sincerely,

Xiuli YANG
