# Supplementary material for: Machine learning for early prediction of the infection in patients with urinary stone after treatment of holmium laser lithotripsy
Source: PLoS One. 2025 May 16;20(5):e0317584. doi: 10.1371/journal.pone.0317584 (PMC12084040; doi:10.1371/journal.pone.0317584)
Supplement: Attachment — Submitted filename: Response to Reviewers2.docx [file pone.0317584.s004.docx]

Dear Editor and Reviewers,

Thank you so much for your comments concerning our manuscript entitled “Machine learning for early prediction of the infection in patients with urinary stone after treatment of holmium laser lithotripsy” (ID: PONE-D-24-39210). Those comments are all valuable and very helpful for revising and improving our paper, as well as the important guiding significance to our researches. We have studied comments carefully and have made correction which we hope meet with approval.

Reviewer #1:

1. It's still suggested to calculate PPV although you have reported F1. This performance index could give audience a more comprehensive understanding of the model prediction, and it will not be a big issue if the index is relatively low because it's hard to get both a ideal recall and PPV in the same clinic predition model.

**Authors' Response**: Thanks for the kind and insightful comments and suggestions! We have added the PPV index to evaluate our model. Changes to our manuscript were all highlighted within the document by using red-colored text.

Yours sincerely,

Xiuli YANG
